# Towards a neurobiology of early intervention: the importance of critical periods for preventive psychiatry and youth mental health

**DOI:** 10.1038/s41380-026-03734-w

**Published:** 2026-07-29

**Authors:** Peter J. Uhlhaas

**Affiliations:** 1 Department of Child and Adolescent Psychiatry, Charité Universitätsmedizin, Berlin, Germany; 2 Einstein Center for Youth Mental Health (ECYM), Berlin, Germany; 3 German Center for Mental Health (DZPG), partner site Berlin-Potsdam, Berlin, Deutschland

**Keywords:** Neuroscience, Psychiatric disorders

## Abstract

Evidence has accumulated that youth, the period between 12–25 years, may constitute a crucial period for the early detection and intervention of mental disorders. Thus, there is emerging evidence that targeted psycho-social and pharmacological interventions have potential to improve clinical outcomes in at-risk individuals as well as in youths with a first-episode of mental illness. However, while support for the early intervention paradigm has gained momentum, an explanatory framework for the emergence of mental disorders as well as for the mechanisms of preventive interventions is currently not available. Here, I will outline a neurobiology of early intervention that will highlight the importance of linking emerging psychopathology with developmental neuroscience and systems biology. Specifically, I will propose that critical periods during late brain development in combination with environmental exposures could account for the elevated occurrence of psychosis, affective disorders, substance abuse and personality disorders. In addition, the neurobiological mechanisms underlying critical periods could be important for understanding the potential of preventive interventions and the identification of biomarkers. Together, a neurobiological grounding of early intervention could provide a conceptual and empirical framework to advance early detection and prevention of mental disorders during youth.

## Mental disorders, early intervention and neurobiology

Until recently, the focus to improve clinical outcomes of mental disorders has been on fully established disorders in adulthood while early intervention and prevention have been relatively neglected [1]. However, there has been recently a shift towards early intervention of emerging mental disorders during youth [2], the period between 12–25 years, which is motivated by epidemiological evidence that the majority of mental disorders begin before the age of 25 [3].

The early intervention paradigm was initially developed to reduce the duration of untreated psychosis (DUP) [4], providing a blueprint for pre-emptive and illness-course modifying interventions in psychiatry [1]. The introduction of clinical high-risk for psychosis (CHR-P) criteria then enabled a systematic approach to secondary prevention [5] based on the observation that the onset of psychosis in the majority of cases is preceded by a prodromal period [6]. Subsequently, the early intervention paradigm has been extended to bipolar disorder [7], eating disorders [8] and borderline personality disorder [9], given similar developmental trends in age of onset and symptom expression [3]. However, despite the potential of the early intervention approach, significant challenges remain, including questions around stigma and labelling in at-risk populations [10], the potential harmful effects of preventive interventions [11] and ethical implications. Moreover, a framework for explaining the increased incidence of mental disorders as well as for the mechanisms and rationale of preventive interventions is currently not available [12].

**Fig. 1:**
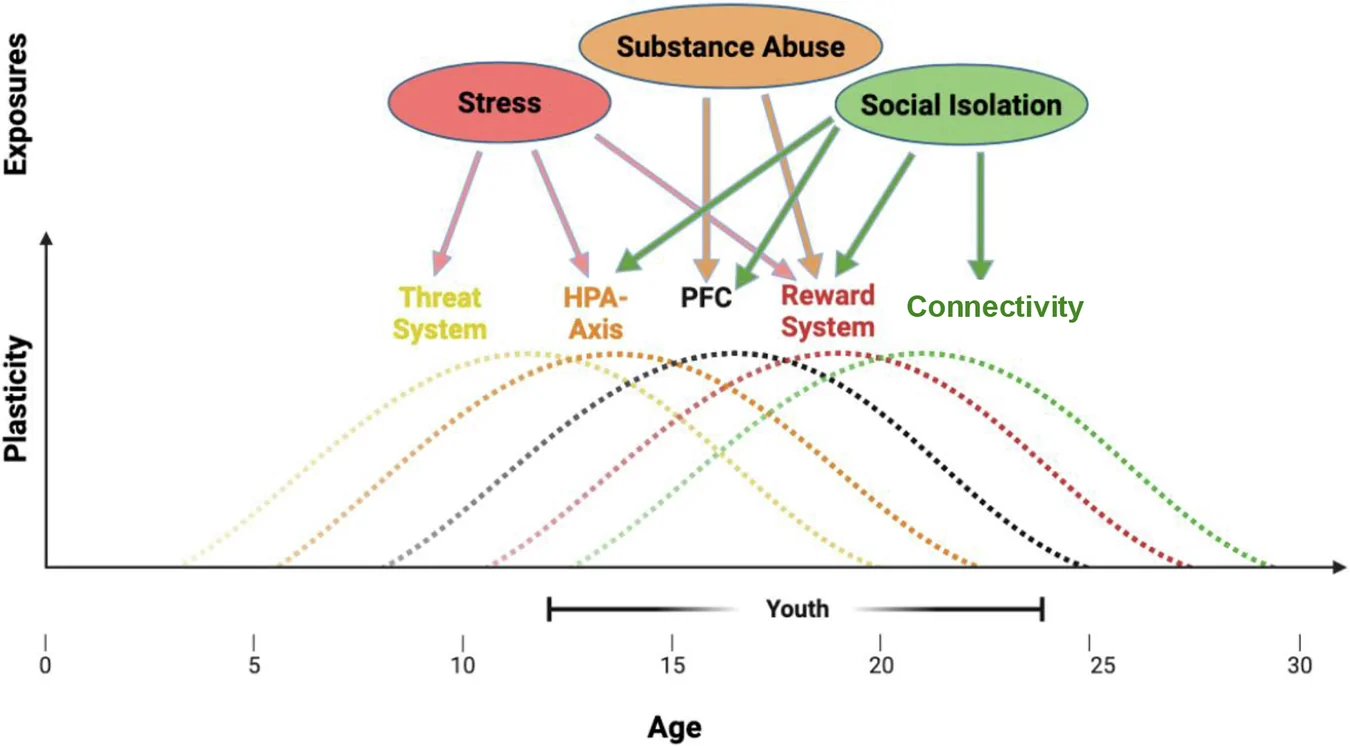
Overview of critical periods during brain development and their interactions with risk factors during youth. Curves indicate the plastic potential for different neural systems between 0 and 30 years of age: threat-regulation involving cortical-hippocampal-amygdala circuits HPA-axis system PFC subsumes local changes in PFC-properties (E/I-balance, Dopamine) Reward System comprising striatum and PFC and Long-Range Connectivity Top panel: displayed are risk-factors, such as stress, substance abuse and social isolation that have been shown alter permanently circuit properties during critical periods during youth. HPA-axis hypothalamic-pituitary-adrenal axis, PFC prefrontal cortex, E/I balance Excitation/Inhibition-balance, PFC prefrontal cortex.

To address these questions, I will outline a “neurobiology of early intervention” that draws on recent findings in developmental neuroscience, highlighting the unique properties of neural circuits during late brain development. In the first part, I will outline evidence that “critical periods” (CPs) provide unique windows of vulnerability due to the elevated plasticity of neural circuits that could account for the development of ill-mental health during youth (Fig. 1). In the second part, I will propose that the timing and mechanisms of CPs may be relevant for reducing the duration of untreated illness (DUI) and preventive interventions in at-risk populations. Finally, I will explore the importance of CPs for the development of novel treatments and biomarkers.

## Why do mental disorders emerge during youth?

“Mental disorders are chronic diseases of the young.” [13]

### Critical periods and psychopathology

A central conundrum is the preferential manifestation of mental disorders during youth. Approx. 75% of all major mental disorders beginning before the age of 25 and the peak age of onset of any mental disorders is 14.5 years [3], with ill mental-health being the leading cause of disability in this age group [2].

Almost 20 years ago, Paus et al. [14] outlined emerging evidence that modifications in neural systems during adolescence^1^ could be relevant for understanding ill mental health, suggesting that “…many questions about adolescent brain development and its impact on disease can best be investigated in animal models (p 955).” Indeed, recent advances in neuroscience have provided evidence on the importance of CPs during late brain development that could provide a novel framework for addressing the preferential manifestation of mental disorders a during youth.

CPs are key principle in developmental neuroscience that describe windows during brain maturation when environmental input is required for the proper development of a neural circuit. CPs encompass periods of heightened plasticity that involve substantial, rapid changes to neural circuits whereby neural responsiveness is elevated to specific exposures after which additional tuning is reduced [15]. CPs were first identified in visual cortex [16] through demonstrating that occlusion of one eye during early in life causes a persistent loss of visual acuity, followed by evidence for CPs in other sensory systems [17,18].

The neurobiology of CPs has been identified with increasing detail in recent years [18, 19], suggesting a much more dynamic and temporally extended process than previously assumed. In particular, the balance between excitation and inhibition (E/I-balance) in neural circuits has emerged as an important factor as a subclass of gamma-aminobutyric acid (GABA) neurons, so-called parvalbumin-expressing (PV+) GABAergic interneurons, are involved in triggering CPs [20]. Opening of CPs is furthermore related to intrinsic as well as cell-extrinsic factors, such as brain-derived neurotrophic factor (BDNF) [21]. In addition, sensory experience, stress, genetic and epigenetic factors contribute towards CP-timing [19].

Following the adjustment of E/I-balance, several molecular mechanisms, such as perineuronal nets (PNNs), myelin- and nicotinergic receptors, consolidate plastic changes [22]. Interestingly, timing of CPs can be altered and even reopened in the adult cortex, for example through pharmacological manipulations, such as psychedelics [23].

### Evidence for critical periods in neural systems relevant for psychopathology

A hallmark of the period between 12-25 years is the pruning of synaptic connections [24], which is accompanied by the ongoing myelination of intracortical fibers (25), which stabilize neuronal circuits through reducing neuronal plasticity [19]. As a result, the volume of gray matter (GM) decreases while whiter matter (WM) increases. Recent findings have shown that GM/WM modifications follow a hierarchical sensorimotor-to-association axis [25], consistent with the temporal patterning of CPs across cortical regions [19].

Further evidence for CPs comes from studies that investigated neural systems that are relevant for emerging psychopathology (Table 1). Threat regulation is the ability to modulate physiological, affective, and cognitive responses to perceived danger and uncertainty, and is crucially involved in anxiety disorders that typically peak during adolescence [26]. Among the neural circuits involved, the amygdala, hippocampus and PFC are of particular interest, which undergo major modifications during this developmental period.

**Table 1:** Critical Periods of Core Neural Systems during Youth.

	Threat Regulation	HPA-Axis	PFC	Reward System	Long-Range Connectivity
Development					
Human	Early adolescence	Adolescence	Adolescence, Early Adulthood	Adolescence, Early Adulthood	Adolescence, Early Adulthood
Rodent	P25-45	P25-60	P30-60	P30-60	P25-60
Circuits/Brain Regions	Amygdala, PFC	HPA, HP, Amgydala	DLPFC, mPFC	VTA, PFC, Amygdala, HP	PFC-Thalamus, PFC-Parietal, PFC-HP, PFC-Amygdala
Behaviour/Cognition	Fear extinction, retention, responding	Stress response	WM, Inhibition, Executive Functions	Reward Processing, Decision Making	Emotion Regulation, Attention, Reward Processing
Molecular Mechanisms	PNNs, Nogo Receptors, Lynx1,	-	PV+Interneurons, BDNF,	D1/D2 Receptors,	Myelination, Lynx1
Exposures/Risk-Factors	Stress	Social Isolation, Stress	Social Isolation, Cannabis	Substance Abuse, Stress, Social Isolation	Social Isolation

On the behavioral level, extinction learning is reduced during adolescence [27] as well as contextual threat responding [26] but this has not been observed in all studies [28]. Several CP-mechanisms have been demonstrated to underlie developmental modifications in extinction learning, including PNNs as well as Nogo receptors and Lynx1 expression [29]. In addition, exposure to stress during adolescence can have both long- and short-term effects on amygdala-PFC circuits [30], including an accelerated development of amygdala–PFC connectivity [31].

The stress response has evolved to maintain homeostasis under conditions of real or perceived stress, which is mediated by the hypothalamic–pituitary–adrenal (HPA) axis. HPA-axis dysregulation has been implicated in psychosis, affective disorders and personality disorders [32]. Previous findings have highlighted that early development extending to the first months of life is a CP for establishing set-points for the reactivity and regulation of the HPA-system [33].

This perspective has been complemented by recent evidence that the HPA-axis during youth undergoes major modifications [34]. Thus, a variety of stressors are associated with elevated and prolonged HPA-axis responses in youth compared to adulthood [35]. Moreover, adolescent rodents are characterized by reduced habituation to chronic stress exposure compared to adult animals [36]. On the other hand, data from institutionalized children who had a supportive caregiving during adolescence have shown HPA-axis activity similar to children who were never exposed to early deprivation [37]. Finally, social isolation can profoundly dysregulate HPA-axis activity during adolescence, causing depressive- and anxiety-like traits as well as cognitive impairments in mice, which are linked to altered HPA axis activity [38].

The development of higher cognitive functions mediated by the PFC during youth has been linked CP mechanisms [39], which could be relevant for schizophrenia, depression, and bipolar disorder. Microglia undergo time-limited modifications in the PFC during adolescence and induction of transient and cell-specific deficiency of microglia induces PFC-associated impairments in cognitive functions, dendritic complexity and synaptic structures in adulthood [40].

At the behavioral level, developing executive control, response inhibition and working memory [39] are accompanied by profound changes of dopaminergic, glutamatergic, and GABAergic receptors in the PFC during youth. Thus, expression levels of PV+ interneurons increase [41] while downregulation during adolescence leads to permanent changes in E/I-balance [42]. In addition, dopaminergic modulation of GABAergic interneurons matures during adolescence [43] as well as interactions between dopamine and NMDA-Rs [44]. Finally, expression levels of BDNF together with PNNs increase [45].

Several exposures alter the properties of neural circuits in the PFC consistent with a CP during youth. Thus, social isolation in juvenile mice leads to a specific deficit of PV+ interneurons which in turn are necessary for adult social behavior to develop [46]. Similar observations have been made on the impact of social isolation on dendritic complexity and BDFN-levels [47] and E/I-balance in the PFC [46, 48].

Reward processing involves the ability to perceive, evaluate and respond to rewarding stimuli and has been closely linked to the integrity of the striatum and PFC, which are critically involved in schizophrenia, affective disorders and substance abuse [49]. Dopamine synthesis peaks during adolescence [50] as well as D1 and D2 receptors in the caudate and putamen [51], which are accompanied by the strengthening of dopaminergic projections to the medial PFC [52].

Youths are characterized by increased levels of sensation seeking and risk taking, particularly during the presence of peers [53], which affect the self-image more than during other developmental periods [54]. Heightened social reward learning during adolescence in the mice was recently shown to be regulated by the interaction of oxytocin system and sub-cortical reward circuitry and this window can be reopened by an administration of 3,4-methylendioxy-N-methylamphetamin (MDMA) [55].

Risky decision-making is associated with substance abuse during youth and the likelihood of developing substance use disorder is highest if initiated before age 14 [56]. Thus, cocaine exposure during adolescence but not adulthood is associated with chronic attenuation of GABAergic activity and PV+ expression levels in the medial PFC [57]. In addition, binge-like alcohol exposure in adolescent rats reduces adult hippocampal, thalamic, striatal and cortical volumes [58].

Cannabis has been causally linked to emergence of psychosis if initiated during adolescence [59, 60]. This age group is also more sensitive to the effects of Delta-9*-*tetrahydrocannabinol (Δ9-THC) and cannabinoids [61], (CBR 1) which coincides with the higher levels of cannabinoid receptor 1 during adolescence [62]. In rodents, chronic CB1R agonist exposure during adolescence leads to PFC-dependent cognitive changes that has been linked to the suppression of GABAergic neurotransmission in the PFC [63], resulting in downstream effects on glutamate and dopamine release [64], suggesting a CP for the effects of cannabinoids [61].

Finally, stress exposure during adolescence leads to a permanent dysregulation of midbrain dopaminergic neurons which is associated with a loss of PV+ interneurons and their associated PNNs, a phenotype consistent with observations in schizophrenia [65]. Importantly, removing PNNs in adult animals from the ventral hippocampus before stress exposure caused cognitive and social interaction impairments and a hyperdopaminergic state in the ventral tegmental area [66].

Modifications of local circuits are accompanied by an extensive integration with brain networks through long-range connections during youth that are fundamental for the maturation of behavioral and cognitive milestones. A fundamental feature of brain networks are “hubs”, which form a dens core that enhances the functional integration of anatomically distributed and specialized neural systems [67]. Adolescence involves a selective strengthening of frontal–subcortical and frontal–parietal hub–connectivity, which may confer improved control of the PFC over subcortical and cortical regions [68], leading to enhanced emotion regulation, reward processing and cognitive control. Thus, the selective strengthening of PFC-amygdala connectivity [69] may be particularly relevant for emotion regulation during adolescence [70] but this this pattern has not been observed consistently [71].

In addition, transient thalamic inhibition in mice between P20-P50 is associated with lasting changes in the PFC in adulthood, including deficits in cognition, decreased excitatory drive in the PFC as well as reduced anatomical thalamo–medial PFC inputs [72]. Similar effects on PFC-properties have been observed as the result of juvenile social isolation [48]. Finally, chemogenetic suppression of top-down activity during adolescence, but not later periods, produces long-lasting visual attentional behavior which is mediated by the expression of a nicotinic brake Lynx1 [73].

## Preventive interventions and critical periods

### Critical periods and the timing of interventions

Elevated plasticity of CPs during youth could not only provide a biological vulnerability together with environmental exposures for the development of mental disorders but also offer windows for treating and correcting developmental aberrations through targeted interventions.

An important objective of the early intervention approach is the reduction of DUI [74], which was first proposed in the context of psychosis [4]. Extended DUIs ranging from 2–10 years [2] have been demonstrated for obsessive compulsive disorder [75], bipolar disorder [76] and eating disorders [77], that in turn have been linked to poorer clinical outcomes and trajectories. However, the underlying neurobiological mechanisms and effects of DUI on clinical outcomes remain unclear [12].

Initially, prolonged DUP in schizophrenia was considered to have “toxic effects” through an unknown neurologic or psychological mechanism [78]. One possibility is that the consequences of DUP may be mediated by CP parameters. Smith et al. [79] observed that schizophrenia patients with a shorter DUI were characterized by increased expression of genes associated with CPs compared to those with a longer DUI. In addition, reduced BDNF-levels as well as PNNs, which have been implicated in closing CPs [21, 80], are reduced in a schizophrenia patients that correlate with cognitive deficits and prolonged DUP [81]. Similar data have been reported in bipolar disorder where a longer DUI has been linked to disturbances in neuroplasticity [82].

The timing of CPs may also be important for preventive interventions. Indicated prevention has so far focused on CHR-P populations and both pharmacological as well as psycho-social interventions have been investigated [83]. In addition, emerging evidence is available for studies into CHR-BP populations as well as youths at-risk for depression [1]. However, the most recent meta-analysis concluded that there was insufficient evidence for a robust effect in CHR-Ps [83].

This contrasts with data from genetic and pharmacological animal models of schizophrenia which target circuit and behavioral deficits across development. Thus, cognitive training [84], pharmacology [85, 86] brain stimulation [87], and activation of PV+ interneurons [88] resulted in improved and sometimes even a complete reversal of profound circuit and behavioral dysfunctions. However, the effects across studies were closely linked to a timing of interventions during adolescence while the same behavioral and pharmacological approaches in adult animals had either limited or no effect.

One possibility is that the discrepancy between animal models and human studies can be explained by differences in circuit properties. However, mechanisms and timing of CPs are largely conserved [18] and overlapping CP-mechanisms and properties have been demonstrated [27]. An alternative explanation is that current interventions in CHR-P populations may occur during windows with limited CP-plasticity. Indeed, the majority of clinical trials in CHR-Ps have been conducted in adult populations [83].

### Towards developmentally sensitive interventions

Currently, interventions for youths are essentially derived from adult populations despite considerable differences in anatomy, physiology and functional properties of neural circuits. Accordingly, a paradigm shift may be required that incorporates CP-mechanisms for the identification of novel targets and interventions.

Interventions for anxiety are increasingly informed by the differences in diminished fear extinction and retention of learned safety across development [89]. Thus, reduced fear extinction during adolescence may have important implications for exposure therapies (19). Recent developments have also seen the adaptation of modified psychotherapeutic interventions for borderline personality disorders for adolescent populations [90].

Moreover, novel pharmacological approaches have been proposed that involve CP-mechanisms. PNNs act as a molecular brake on synaptic plasticity [91] and their degradation has been implicated in schizophrenia as well as stress-related disorders [92]. Accordingly, enhancing PNNs to promote neuroplasticity during adolescence may be relevant for preventive interventions [93]. Indeed, a recent study showed that cannabidiol reverses loss of PV+ interneurons and PNNs in a mouse of model of schizophrenia [94].

A common feature of psychedelics is their impact on CPs [23]. Psychedelics have been shown to reopen social reward learning in adulthood in mice [95] and Ketamine reactivates adult cortical plasticity to restore vision in amblyopia which is consistent with the effects on PFC spinogenesis [96]. Together, these data highlight that psychedelics reopen CPs that could enhance psycho-social and pharmacological interventions [23]. The therapeutic administration of psychedelics continues to face barriers, however, in particular in younger populations [97].

### Sensitive periods and biomarkers

Clinical trials investigating preventive interventions in at-risk populations are likely to require stratification strategies given the highly heterogeneous clinical phenotypes [83]. This is particularly important given that many at-risk individuals will not develop a chronic mental disorder [2]. Recent advances in neuroimaging and computational modelling have provided first proxy measures for CPs that could be harnessed for stratification and early detection [98]. Myelination is an important factor in CP-plasticity which can be quantified using maps derived from T1-weighted (T1w) and T2-weighted (T2w) MRI data and changes T1/T2 ratios are consistent with the temporal patterning of CPs [19] and E/I-balance parameters [99] that can be derived from EEG/MEG-data [19]. Finally, biophysical models in combination with resting-state fMRI-data have demonstrated differences in E/I ratio during brain development that can be harnessed to assess differences across the cortical hierarchy [100].

## Towards a neurobiology of early intervention

Converging evidence from systems neuroscience highlights that core neural circuits involved in psychopathology are characterized by CPs during youth that together with exposures to risk factors could lead to lasting changes in circuit properties and therefore to the emergence of clinical phenotypes associated with psychosis, affective disorders, substance abuse and personality disorders. However, the association between environmental exposures and mental disorders supporting the notion of CPs has been challenging to demonstrate in epidemiological studies [101, 102].

One possibility is that the effects of environmental exposures are mediated by genetics as well as through interactions with other risk factors, which require novel approaches to unravel the complex interplay between environment and brain development [103]. In addition, it is likely that emerging mental disorders do not follow a linear relationship between exposures and symptomatic manifestations. Neural and behavioral compensatory mechanisms as well as the complex interactions with the environment may lead to non-linear development trajectories that can either accelerate or delay the emergence of subthreshold and manifest mental illnesses during youth.

The evidence on CPs may have several implications for current research into early interventions. Firstly, CPs during late brain development should be an important guiding principle to motivate early intervention and prevention during youth. In addition to clinical and economical imperatives, the evidence on CPs could constitute a key scientific principle that supports, for example, the reduction of DUI across a range of mental disorder, as plasticity mechanisms are likely to be involved in poor treatment responses and clinical outcomes.

Secondly, current preventive interventions and clinical trials should target younger populations (<18 years of age), given converging evidence from animal models that the timing of treatments may have a crucial impact on the possibility to improve behavioral and circuits deficits. While scientifically and ethically challenging, the current impasse for preventive interventions in CHR-P populations [83] provides a strong motivation to focus on the developing brain that could provide unique “windows of opportunity” to alter developmental trajectories.

Thirdly, interventions for youths require a greater consideration for the neurobiology of CPs. Current treatments are based on research into adult populations without adequate consideration of the differences in behavioral and circuit properties across development. Thus, future research should consider testing developmentally adapted interventions that exploit CPs mechanisms and principles. In addition, the neurobiology of CPs may also provide novel targets for preventive psychiatry.

Fourth, biomarkers based on CPs could be a key component for a precision-based approach through stratifying individuals according to their developmental trajectories. For this approach to succeed, improved non-invasive biomarkers for CPs are required together with normative data to determine deviations from developmental trajectories that could facilitate targeted interventions. This is particularly important for improving the effectiveness of interventions but also reducing the potential harmful effects by targeting those individuals with a substantial risk to develop a serious mental illness.

Together, a “neurobiology of early intervention” could provide an empirical framework bringing together developmental neuroscience, epidemiology and psychiatry that could further advance early detection and prevention of serious mental illnesses which remains a fundamental challenge for the field.

## Footnotes

1 During human development, adolescence refers to the period between 12-21 years which can be further subdivided into early adolescence (10-13 years), mid-adolescence (14-17 years) and late adolescence (18-21 years). In rodents, adolescence is typically defined as the entire postnatal period ranging from weaning (postnatal day (PND) 21) to adulthood (PND 60). Moreover, the period can be also divided into 1) early adolescence (prepubescent or juvenile, PND 21 – 34), middle adolescence (peri-adolescent, PND 34 – 46), and late adolescence (young adult, PND 46 – 59).
